# Accurate and rapid screening model for potential diabetes mellitus

**DOI:** 10.1186/s12911-019-0790-3

**Published:** 2019-03-12

**Authors:** Dongmei Pei, Yang Gong, Hong Kang, Chengpu Zhang, Qiyong Guo

**Affiliations:** 1 Department of Family Medicine, Shengjing Hospital, China Medical University, Shenyang, Liaoning China; 2 University of Texas Health Science Center at Houston, Houston, Texas USA; 3 Department of radiology, Shengjing Hospital, China Medical University, Shenyang, Liaoning China

**Keywords:** Data mining, Diabetes, Screening

## Abstract

**Background:**

Prediction or early diagnosis of diabetes is crucial for populations with high risk of diabetes.

**Methods:**

In this study, we assessed the ability of five popular classifiers (J48, AdaboostM1, SMO, Bayes Net, and Naïve Bayes) to identify individuals with diabetes based on nine non-invasive and easily obtained clinical features, including age, gender, body mass index (BMI), hypertension, history of cardiovascular disease or stroke, family history of diabetes, physical activity, work stress, and salty food preference. A total of 4205 data entries were obtained from annual physical examination reports for adults in the Shengjing Hospital of China Medical University during January–April 2017. Weka data mining software was used to identify the best algorithm for diabetes classification.

**Results:**

The results indicate that decision tree classifier J48 has the best performance (accuracy = 0.9503, precision = 0.950, recall = 0.950, *F*-measure = 0.948, and AUC = 0.964). The decision tree structure shows that age is the most significant feature, followed by family history of diabetes, work stress, BMI, salty food preference, physical activity, hypertension, gender, and history of cardiovascular disease or stroke.

**Conclusions:**

Our study shows that decision tree analyses can be applied to screen individuals for early diabetes risk without the need for invasive tests. This procedure will be particularly useful in developing regions with high epidemiological risk and poor socioeconomic status, and enable clinical practitioners to rapidly screen patients for increased risk of diabetes. The key features in the tree structure could further facilitate diabetes prevention through targeted community interventions, which can potentially improve early diabetes diagnosis and reduce burdens on the healthcare system.

## Background

The worldwide incidence of diabetes rose from 108 million in 1980 to 422 million in 2014, and could potentially be the seventh-leading cause of death in 2030 [1]. However, half of the patients with diabetes are unaware of their disease. The incidence of diabetes (100 million adult patients) in China was the highest worldwide in 2015, whereas 52.7% of these patients (50 million) are undiagnosed [2, 3]. Hence, early detection and prevention of diabetes is a severe challenge in China.

The American Diabetes Association recommends annual screening for diabetes in patients older than 45 years and in younger patients with major risk factors [4]. China’s National Plan for Non-Communicable Diseases Prevention and Treatment (2012–2015) identified diabetes as one of the priority diseases in China, and proposed several recommendations to predict diabetes based on blood glucose tests and routine physical examinations [5].

The main challenge in screening for diabetes is economic, including expensive blood work and additional human labor, which is even more challenging in developing countries [6]. The World Health Organization recommends that simple strategies should be developed to identify patients with risk for diabetes and then implement early lifestyle interventions [7]. To achieve these recommendations, it is crucial to develop a simple and accurate diabetes screening method.

Developing appropriate disease prediction algorithms can be technically challenging. In a Brazilian investigation, Lélis et al. [6] applied seven classification techniques to make a diagnosis of meningococcal meningitis and demonstrated this model is accurate and affordable. Choi et al. [8] developed two models to screen for prediabetes of 9251 individuals using an artificial neural network (ANN) and support vector machine (SVM) and performed a systematic evaluation of the models using internal and external validation, and concluded that the SVM model is superior to the ANN model in the screening for prediabetes. In another Brazilian study, Olivera et al. [9] utilized and compared machine-learning algorithms to develop predictive models using data from ELSA-Brasil and found that most of these predictive models yielded similar results and demonstrated the feasibility of identifying individuals with highest risk of having undiagnosed diabetes through easily-obtained clinical data. Data mining and machine learning are analytical methods that leverage artificial intelligence to identify patterns in large data sets, make decisions with minimal human intervention, and build models. There is considerable interest in determining how different classification techniques from machine learning can be utilized as disease prediction tools [10–19]. These tools have been used to diagnose diabetes [3, 8–10, 20–22], meningitis [6], glaucoma [11], asthma [12], coronary artery disease [13], cancer [14–17, 23], tuberculosis [18, 24], hypertension [25], and heart arrhythmia [26].

The objective of this study is to use easily obtained and directly observable clinical data to construct a predictive model to identify patients with increased risk for diabetes. Specifically, we utilize data mining and machine learning to develop an accurate diabetes classifier that can rapidly screen clinical data. Our approach will be particularly useful in locations with high epidemiological risk and poor socioeconomic status, where patients cannot afford medical laboratory costs [6]. Rapid identification of patients with high diabetes risk can help to avoid disease progression and prevent the incidence of disease complications.

## Methods

### Study population

A total of 8452 annual physical examination reports between January 2017 and April 2017 were collected from the electronic health records database in Shengjing Hospital of China Medical University, located in the center of Liaoning Province in China. We adopted the nine most frequently used features from previous studies of diabetes prediction models [8, 20, 27–30]. These features are either directly observable or easily obtained without expensive and invasive tests. Approval for this study was obtained from the Shengjing Hospital (reference number 2017PS42K).

**Fig. 1:**
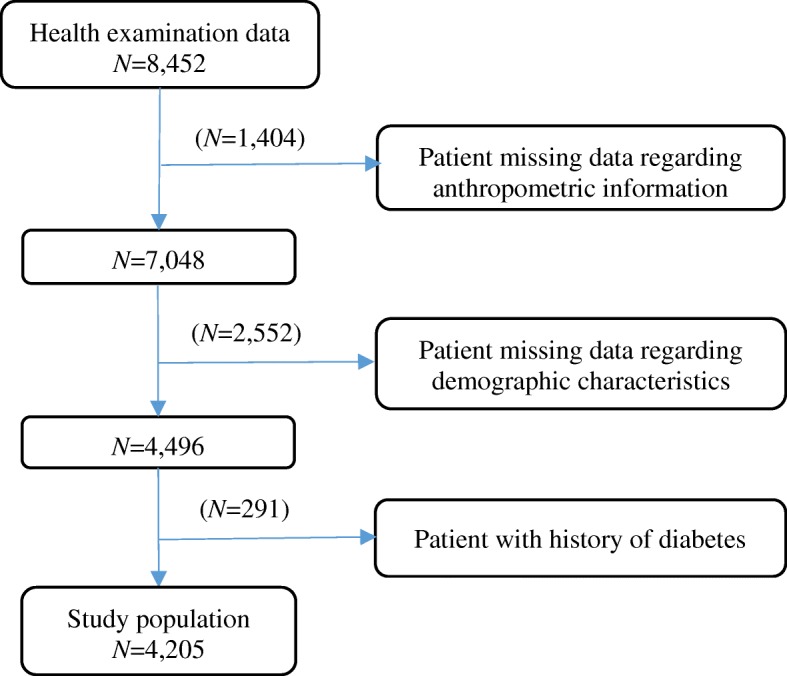
Flow chart of records that were excluded from the physical examination database of Shengjing Hospital of China Medical University (January–April, 2017)

The nine features included age, gender, body mass index (BMI), hypertension, history of cardiovascular disease or stroke, family history of diabetes, physical activity, work stress, and salty food preference (eating the salty meat or fish 4–7 times a week). Among 8452 records, a total of 3956 records were excluded due to missing data for BMI, blood pressure, family history of diabetes, history of cardiovascular disease or stroke, physical activity, work stress, or salty food preference. Records with past history of diabetes (291 records) also were excluded because we focused on predicting prediabetes and diabetes. Finally, a total of 4205 records were included in this study as shown in Fig. 1.

### Data collection and transformation

The nine features were characterized for data analysis. Age and gender were demographic characteristics. Family history of diabetes was defined as any family member previously diagnosed by a physician as diabetic or prediabetic (Yes = 1, No = 0). BMI was calculated as body weight divided by the square of height in meters and BMI ≥ 25 was defined as overweight. History of cardiovascular disease or stroke was defined as the patient previously diagnosed with coronary heart disease or stroke by a physician (Yes = 1, No = 0). Physical activity indicated if the patient engaged in more than 30 min of exercise 3 days a week (More = 1, Less = 0). Work stress was grouped into three levels according to the patients’ subjective impression (High = 2, Moderate = 1, Low = 0). Salty food preference (salty meat or fish) indicated if the person preferred salty food for 4–7 days a week (Yes = 1, No = 0).

BMI and hypertension were defined and measured as below. BMI was calculated as weight in kilograms divided by the square of height in meters (kg/m2); BMI ≥ 25 was defined as overweight. Hypertension was defined as systolic blood pressure ≥ 140 mmHg, or diastolic blood pressure ≥ 90 mmHg, and/or use of medication for blood pressure control.

Each report included a diagnosis (diabetes or normal) based on fasting plasma glucose. Diabetes diagnoses included prediabetes and type 2 diabetes, and was defined as fasting plasma glucose ≥5.6 mmol/L [8, 20].

### Variable characteristics

After data preparation and transformation, the final database consisted of 4205 records and 10 variables. These 10 variables included 9 input variables and one target variable. The target variable consisted of two classes: one class was the diagnosis of diabetes, the other class was normal. The characteristics of participants and chi-square test results between two groups are presented in Table 1. There were statistically significant differences in the nine features between the two groups, at a significance level of 0.05.

**Table 1:** Characteristics of variables in diabetes and normal groups

Variable	Possible values	Diabetes *N* = 709	Normal *N* = 3496	*p*-value	χ^2^ test
Age	20–34 years old	36 (5.1%)	1718 (49.1%)	< 0.001	269.33
	35–49 years old	207 (29.2%)	1246 (35.7%)		
	50–65 years old	466 (65.7%)	532 (15.2%)		
Gender	Male	348 (49.1%)	1123 (32.1%)	< 0.001	16.25
	Female	361 (50.9%)	2373 (67.9%)		
Body mass index	< 25	250 (35.3%)	2806 (80.3%)	< 0.001	18.87
	≥25	459 (64.7%)	690 (19.7%)		
Hypertension	Yes	221 (31.2%)	755 (21.6%)	< 0.001	15.22
	Non-hypertension	488 (68.8%)	2741 (78.4%)		
Salty food preference	No	384 (54.2%)	2598 (74.3%)	< 0.001	9.33
	Yes	325 (45.8%)	898 (25.7%)		
History of cardiovascular disease or stroke	No	627 (88.4%)	3190 (91.2%)	0.018	122.25
	Yes	82 (11.6%)	306 (8.8%)		
Family history of diabetes	No	335 (47.2%)	3133 (89.6%)	< 0.001	154.21
	Yes	374 (52.8%)	363 (10.4%)		
Physical activity	Less	542 (76.4%)	2043 (58.4%)	< 0.001	33.68
	More	167 (23.6%)	1453 (41.6%)		
Work stress	Low	129 (18.2%)	1054 (30.2%)	< 0.001	81.54
	Moderate	353 (49.8%)	1993 (57.0%)		
	High	227 (32.0%)	449 (12.8%)		

### Classifier comparison

We applied five popular classifiers to train the dataset, including J48 (class for generating a pruned or unpruned), AdaboostM1 (method for boosting a nominal class classifier), SMO (implements John Platt’s sequential minimal optimization algorithm for training a support vector classifier), Bayes Net (Bayes network learning method that implements a hill climbing algorithm restricted by an order on the variables), and Naïve Bayes (class for a naïve Bayes classifier using estimator classes). Weka software (version 3.8; University of Waikato, Hamilton, NZ) [6, 16] was used to assess the classifiers and identify the best algorithm for diabetes classification. To avoid over-fitting and unnecessary complexity, the decision tree created by the J48 algorithm was pruned by removing nonessential terminal branches. This pruning method was based on defined algorithms and did not affect the classification accuracy [6, 21, 25].

### Classifier accuracy and performance evaluation

The entire dataset was randomly divided into two parts: the training set consisted of 70% of the data for model development, and the test set consisted of the remaining data (30%) for model validation [21, 31]. The algorithms were compared based on accuracy, precision, recall, *F*-measure, and the area under the receiver operating characteristic (ROC) curve (AUC), and the best-performing algorithm was selected [21, 32]. Eqs. 1–2 were used to calculate the accuracy, precision, recall, and *F*-measure, respectively.

3 $$ Accuracy=\left( TP+ TN\right)/\left( TP+ TN+ FP+ FN\right) $$

4 $$ Precision= TP/\left( TP+ FN\right) $$

5 $$ Recall= TP/\left( TP+ FP\right) $$

6 $$ F\hbox{--} measure=2/\left(1/ Precision\right)+\left(1/ Recall\right) $$

The AUC summarizes ROC curves by indicating whether the classifier is more likely to distribute the score as positive rather than the randomly selected negative sample. Better models have larger AUC values. The relative accuracy of the classification test is graded according to the following scale [18]: Excellent = 0.90–1; Good = 0.80–0.90; Fair = 0.70–0.80; Poor = 0.60–0.70; Fail = 0.50–0.60.

## Results

**Fig. 2:**
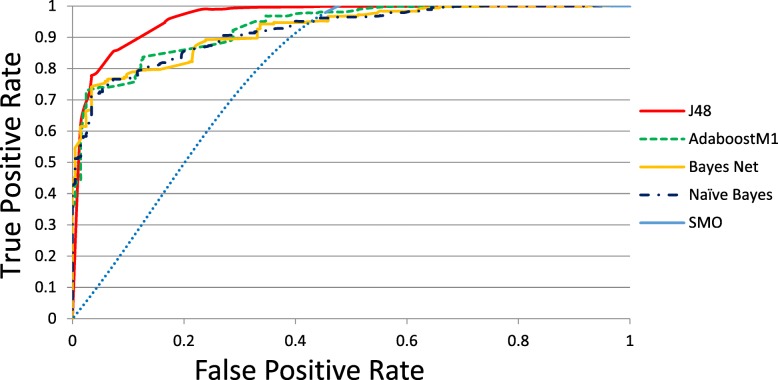
ROC curve of all algorithms

A total of 4205 records (2734 females and 1471 males) were selected for this analysis, which included 709 (16.86%) diabetes diagnoses and 3496 (83.14%) normal patients. Table 2 presents the performance of all classifiers, and shows that J48 exhibits better results than others (accuracy = 0.9503, precision = 0.950, recall = 0.950, F-measure = 0.948, and AUC = 0.964). Figure 2 presents the ROC curves of all classifiers.

**Table 2:** The results of classification algorithms

Model	Accuracy	Precision	Recall	*F*-Measure	AUC
AdboostM1	0.9127	0.908	0.913	0.906	0.933
J48	0.9503	0.950	0.950	0.948	0.964
SMO	0.9078	0.903	0.908	0.900	0.763
Naïve Bayes	0.8934	0.886	0.893	0.888	0.922
Bayes Net	0.8878	0.881	0.888	0.883	0.924

*AUC* the area under the receiver operating characteristic (ROC) curve

**Fig. 3:**
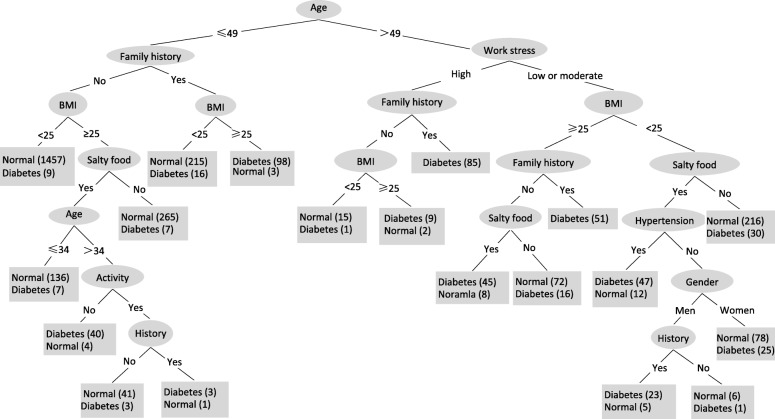
Decision tree of diabetes classifiers. The sample size is given as the number in parentheses at each node

The final tree contains 18 nodes and 19 leaves, as shown in Fig. 3.

The decision tree shows that age was assigned by as the first and most informative node, followed by family history of diabetes, work stress, BMI, salty food preference, physical activity, hypertension, gender, and history of cardiovascular disease or stroke. Most leaves in the left half of the decision tree (≤49 years old) were classified as normal, whereas most leaves in the right half of the decision tree (> 49 years old) were classified as diabetes.

The decision tree can be converted into a set of if-then rules by tracing the path from the root node to each terminal (leaf) node. The if-then rules created by the model are presented in Table 3.

**Table 3:** Nineteen if-then rules extracted from the decision tree in Fig. 3

Rule 1: IF age ≤ 49, without a family history of diabetes, BMI ≤ 25, THEN patient is normal (1457/1466 or 99%)	
Rule 2: IF age ≤ 34, without a family history of diabetes, BMI > 25, prefers salty food, THEN patient is normal (136/143 or 95%)	
Rule 3: IF 35 < age ≤ 49, without a family history of diabetes, BMI > 25, prefers salty food, without physical activity, THEN patient is diabetic (40/44 or 91%)	
Rule 4: IF 35 < age ≤ 49, without a family history of diabetes, BMI > 25, prefers salty food, with physical activity, without history of cardiovascular disease or stroke, THEN patient is normal (41/44 or 93%)	
Rule 5: IF 35 < age ≤ 49, without a family history of diabetes, BMI > 25, prefers salty food, with physical activity, with history of cardiovascular disease or stroke, THEN patient is diabetic (3/4 or 75%)	
Rule 6: IF age ≤ 49, without a family history of diabetes, BMI > 25, without preference for salty food, THEN patient is normal (265/272 or 97%)	
Rule 7: IF age ≤ 49, with a family history of diabetes, BMI ≤ 25, THEN patient is normal (215/231 or 93%)	
Rule 8: IF age ≤ 49, with a family history of diabetes, BMI > 25, THEN patient is diabetic (98/101 or 97%)	
Rule 9: IF age > 49, with work stress high, without a family history of diabetes, BMI ≤ 25, THEN patient is normal (15/16 or 94%)	
Rule 10: IF age > 49, with work stress high, without a family history of diabetes, BMI > 25, THEN patient is diabetic (9/11 or 82%)	
Rule 11: IF age > 49, with work stress high, with a family history of diabetes, THEN patient is diabetic (85 or 100%)	
Rule 12: IF age > 49, with work stress low or moderate, BMI > 25, without a family history of diabetes, prefers salty food, THEN patient is diabetic (45/53 or 85%)	
Rule 13: IF age > 49, with work stress low or moderate, BMI > 25, without a family history of diabetes, without preference for salty food, THEN patient is normal (72/88 or 82%)	
Rule 14: IF age > 49, without work stress high, BMI > 25, with a family history of diabetes, THEN patient is diabetic (51 or 100%)	
Rule 15: IF age > 49, with work stress low or moderate, BMI ≤ 25, prefers salty food, with hypertension, with work stress, THEN patient is diabetic (47/59 or 80%)	
Rule 16: IF age > 49, with work stress low or moderate, BMI ≤ 25, prefers salty food, without hypertension, gender male, with history of cardiovascular disease or stroke, THEN patient is diabetic (23/28 or 82%)	
Rule 17: IF age > 49, with work stress low or moderate, BMI ≤ 25, prefers salty food, without hypertension, gender male, without history of cardiovascular disease or stroke, THEN patient is normal (6/7 or 86%)	
Rule 18: IF age > 49, with work stress low or moderate, BMI ≤ 25, prefers salty food, without hypertension, gender female, THEN patient is normal (78/103 or 76%)	
Rule 19: IF age > 49, with work stress low or moderate, BMI ≤ 25, without preference for salty food, THEN patient is normal (216/246 or 88%)	

## Discussion

In this study, we employed data mining and machine learning to examine the performance of five classifiers (J48, AdaboostM1, SMO, Bayes Net, and Naïve Bayes) and nine non-invasive and easily obtained clinical features (age, gender, BMI, hypertension, history of cardiovascular disease or stroke, family history of diabetes, physical activity, work stress, and salty food preference) for the rapid and accurate identification of individuals with diabetes. The best classifier was trained with the decision tree generated by the J48 algorithm, which had accuracy = 0.9503, precision = 0.950, recall = 0.950, *F*-measure = 0.948, and AUC = 0.964. The results indicate that this strategy successfully achieves accurate and rapid diabetes screening. This approach can be applied for non-invasive prediction of prediabetes and diabetes without the need for expensive lab tests. Thus, this test could be particularly useful in regions with high epidemiological risk and low socioeconomic status.

Decision trees are powerful classification algorithms used in parallel with data mining methods [20, 21, 24, 33, 34]. The first variable (root) in the tree is the most important factor, whereas consecutively distant variables further from the root are ranked in order as less important factors for data classification [21]. This study shows that age is the most important attribute discriminating between those with and without diabetes. Age is followed by family history of diabetes, work stress, BMI, salty food preference, physical activity, hypertension, gender, and history of cardiovascular disease or stroke. These results are consistent with those reported in previous studies [20, 35, 36].

The decision tree shows that family history of diabetes, work stress and BMI are the following important factors after age. The tree identified a subgroup of individuals [1457 patients (99%)] with age ≤ 49, without a family history of diabetes and BMI ≤ 25 that were normal cases. Another subgroup of individuals [98 patients (97%)] with age ≤ 49, with a family history of diabetes, BMI > 25 that were identified as diabetes cases. A subgroup of individuals [85 patients (100%)] with age > 49 and work stress high, with a family history of diabetes were identified as diabetes cases (Table 3). These key features could facilitate diabetes prevention through community interventions. Several large-scale trials have demonstrated the benefits of preventing diabetes with targeted lifestyle interventions [20, 37–39]. By reducing these risk factors, would be rewarded as Therefore, patients who are at a high risk of developing diabetes could be targeted to reduce established risk factors and provide educational programs, which will reduce the public health burden and the number of undiagnosed individuals [8, 40].

A major strength of this study is that we used a real medical dataset of annual physical examinations from Shengjing Hospital of China Medical University. All subjects were subjected to laboratory glucose tests to diagnose prediabetes or diabetes, so the results were more reliable than if the individuals were diagnosed by self-reporting. In 2014, Shengjing Hospital received the Stage Seven award from the Healthcare Information and Management Systems Society for successful implementation of electronic health records and rapid sharing of clinical information via standardized electronic transactions, data warehousing, and data continuity with the emergency department and other ambulatory care departments. Shengjing Hospital routinely collects and stores a large amount of data in the electronic hospital records. We used data mining, machine learning, and knowledge discovery capabilities to identify potential data patterns and specific features containing enough information to increase the accuracy of diabetes predictions [10, 41]. A large-scale study conducted in Iran compared different classification algorithms in the diagnosis of type 2 diabetes and demonstrated that it is therefore highly recommended that the choice and selection of features for data mining applications in disease diagnosis, be done by the help and advice of experts to obtain the best possible results. Artificial neural network is the most accurate method of classification with an accuracy of 97.18% [42].

In the future, we will test the model and develop prediction models with more sensitivity and specificity. We will focus on applying similar methods in different populations using more data. When the amount of data increases, the results will be more robust [17]. Our approach can be extended to larger databases that store more variables and risk factors related to diabetes [22]. The results of these studies could provide novel evidence-based prevention and treatment strategies. Clinical researchers can help to establish new priorities for further analyses by diabetes researchers.

## Limitations

Our study has two limitations. Data were collected from only one large hospital in China. Further studies with additional data from this hospital and other centers need to be performed. This was a cross-sectional design study. The results should be confirmed in a prospective study.

## Conclusion

We utilized data mining classifiers and machine learning to generate a decision tree that identified potential prediabetes and diabetes in clinical data extracted from annual health examination reports in a large Chinese hospital. We assessed the classifiers using nine clinical features that were easily obtained and non-invasive. The J48 classifier had the best performance, and indicates that decision tree analyses can be successfully applied to rapidly and accurately screen for diabetes in clinical practice. This type of work is essential in regions with high epidemiological risk and low socioeconomic status. The tree structure identifies the most important risk factors, and suggests that diabetes prevention programs could be applied through targeted community interventions. This would help improve early diabetes diagnosis and reduce burdens on the healthcare system.

## Abbreviations

AUC: The area under the receiver operating characteristic curve
BMI: Body mass index
SMO: Sequential minimal optimization
